# Profiling of Extracellular Toxins Associated with Diarrhetic Shellfish Poison in *Prorocentrum lima* Culture Medium by High-Performance Liquid Chromatography Coupled with Mass Spectrometry

**DOI:** 10.3390/toxins9100308

**Published:** 2017-09-30

**Authors:** Lei Pan, Junhui Chen, Huihui Shen, Xiuping He, Guangjiu Li, Xincheng Song, Deshan Zhou, Chengjun Sun

**Affiliations:** 1 College of Chemistry and Molecular Engineering, Qingdao University of Science & Technology, Qingdao 266042, China; panleih@outlook.com (L.P.); danny-1218@outlook.com (G.L.); 2 Research Center for Marine Ecology, The First Institute of Oceanography, State Oceanic Administration, Qingdao 266061, China; shenyghui@outlook.com (H.S.); hexiuping@fio.org.cn (X.H.); csun@fio.org.cn (C.S.); 3 Laboratory for Marine Ecology and Environmental Science, Qingdao National Laboratory for Marine Science and Technology, Qingdao, 266071, China; 4 Aquatic Product Quality Inspection Center of Lianyungang, Lianyungang 222001, China; lhs15030471@outlook.com (X.S.); yxm17864266113@outlook.com (D.Z.)

**Keywords:** extracellular toxins, DSP, harmful marine algae, profiling, characterization

## Abstract

Extracellular toxins released by marine toxigenic algae into the marine environment have attracted increasing attention in recent years. In this study, profiling, characterization and quantification of extracellular toxin compounds associated with diarrhetic shellfish poison (DSP) in the culture medium of toxin-producing dinoflagellates were performed using high-performance liquid chromatography–high-resolution mass spectrometry/tandem mass spectrometry for the first time. Results showed that solid-phase extraction can effectively enrich and clean the DSP compounds in the culture medium of *Prorocentrum lima* (*P. lima*), and the proposed method achieved satisfactory recoveries (94.80%–100.58%) and repeatability (relative standard deviation ≤9.27%). Commercial software associated with the accurate mass information of known DSP toxins and their derivatives was used to screen and identify DSP compounds. Nine extracellular DSP compounds were identified, of which seven toxins (including OA-D7b, OA-D9b, OA-D10a/b, and so on) were found in the culture medium of *P. lima* for the first time. The results of quantitative analysis showed that the contents of extracellular DSP compounds in *P. lima* culture medium were relatively high, and the types and contents of intracellular and extracellular toxins apparently varied in the different growth stages of *P. lima*. The concentrations of extracellular okadaic acid and dinophysistoxin-1 were within 19.9–34.0 and 15.2–27.9 μg/L, respectively. The total concentration of the DSP compounds was within the range of 57.70–79.63 μg/L. The results showed that the proposed method is an effective tool for profiling the extracellular DSP compounds in the culture medium of marine toxigenic algae.

## 1. Introduction

Harmful algal blooms occur frequently on a global scale with the deterioration of the marine environment, and pose a serious threat to marine ecosystems, marine aquaculture, and human health [1]. Harmful marine algae generate various toxins, and some can produce diarrhetic shellfish poison (DSP) toxins in global offshore areas. Consumption of shellfish contaminated by DSP toxins may cause stomach spasms, diarrhea, vomiting, and other symptoms [2]. In recent years, human poisoning caused by DSP toxins has occurred worldwide [3,4]. Harmful marine algae that can produce DSP toxins include *Dinophysis* spp. and *Prorocentrum* spp. of the marine dinoflagellates, such as *D. acuminata* [2], *D. acuta* [5], *P. fortii* [6], *P. lima* [7], *P. concavum*, and *P. minimum* [8].

In recent decades, several studies [7,8] have focused on the discovery and characterization of intracellular DSP toxins and derivatives in toxigenic algae. The effect of growth conditions on intracellular DSP toxin accumulation has also been reported [9,10,11,12]. Nevertheless, extracellular DSP toxins released by marine toxigenic algae into the environment have attracted increasing attention in recent years. Nielsen et al. [5] reported that as much as 90% of okadaic acid (OA) and dinophysistoxin-1b (DTX1b) (an analog of DTX1) produced by *D. acuta* are eventually released into the environment as dissolved fraction. Extracellular DSP toxins in the aquatic environment can negatively impact surrounding aquatic organisms. For example, *Dicentrarchus labrax* exposed to a cell-free culture medium is stressed and behaves abnormally, and its gills and liver are impacted [13]. Nauplii of *Artemia salina* die in test solutions containing 50% or more of a cell-free *P. lima* medium within 24 h exposure [14]. To date, only two extracellular DSP toxins (OA and DTX1) have been successfully identified from the culture medium of *P. lima* [2,5,15,16], but complete characterization and quantification of extracellular DSP compounds have yet to be achieved. Objective assessment of the environmental risks of extracellular DSP compounds released by harmful algae cannot be realized due to the absence of comprehensive information of extracellular DSP compounds. Hence, the impact of extracellular DSP compounds on the marine environment is often ignored. Therefore, an effective method for the profiling, complete characterization, and quantification of extracellular DSP compounds in the culture medium of toxigenic algae is necessary for the systematic study of extracellular DSP compounds from different types of harmful marine algae.

With the development of liquid chromatography–mass spectrometry (LC–MS), liquid chromatography–tandem mass spectrometry (LC–MS/MS) [16,17,18] and liquid chromatography–high-resolution mass spectrometry (LC–HR–MS) [19,20,21] have become efficient methods for the rapid identification and determination of DSP toxins in algae and shellfish. Thus, these methods can provide a basis for the complete characterization of extracellular DSP compounds in the culture medium of marine toxigenic algae. In general, the culture medium of toxic marine algae has a high salt content and complex matrix. Thus, developing a suitable enrichment and cleanup method for DSP compounds in the culture medium is necessary. Solid-phase extraction (SPE) and macroporous resin passive adsorption (MRPA) are commonly used for the enrichment and cleanup of lipophilic marine biotoxin [22,23,24,25]. Li et al. [26] successfully enriched three typical DSP toxins in coastal seawater by using SPE. In addition, HP20 macroporous resin was used to enrich extracellular OA and DTX1 in *P. lima* culture medium, and extracellular OA and DTX1 were identified successfully [27,28,29]. To the best of our knowledge, systematic research on the enrichment, identification, and quantitative/semi-quantitative detection of the extracellular DSP compounds in the culture medium of harmful marine algae has yet to be conducted. As a typical producer of DSP toxins, *P. lima* is widely distributed in global coastal seawaters. *P. lima* is easy to cultivate in the laboratory and hence is often used as a model toxigenic alga to study the characteristics of DSP toxin production for harmful marine algae. In the present work, extracellular DSP compounds in *P. lima* culture medium were selected as the target analytes, and a new method for the profiling, complete characterization, and quantification of extracellular DSP compounds in the culture medium of harmful marine algae was developed. The established method was applied in order to characterize and quantify the extracellular DSP compounds in the culture media of different toxigenic algae at different growth stages.

## 2. Results and Discussion

### 2.1. Optimization of Experimental Conditions

In our previous research [30,31,32], LC–MS (Agilent Technologies, Wilmington, DE, USA) was used for the simultaneous separation and detection of multiple lipophilic algal toxins under alkaline mobile phase conditions. On this basis, the LC–HR–MS (Agilent Technologies, Wilmington, DE, USA) and LC–MS/MS (Agilent Technologies, Wilmington, DE, USA) conditions for profiling the extracellular DSP compounds in the culture medium of marine algae were determined by further optimization of the gradient elution programs.

**Figure 1:**
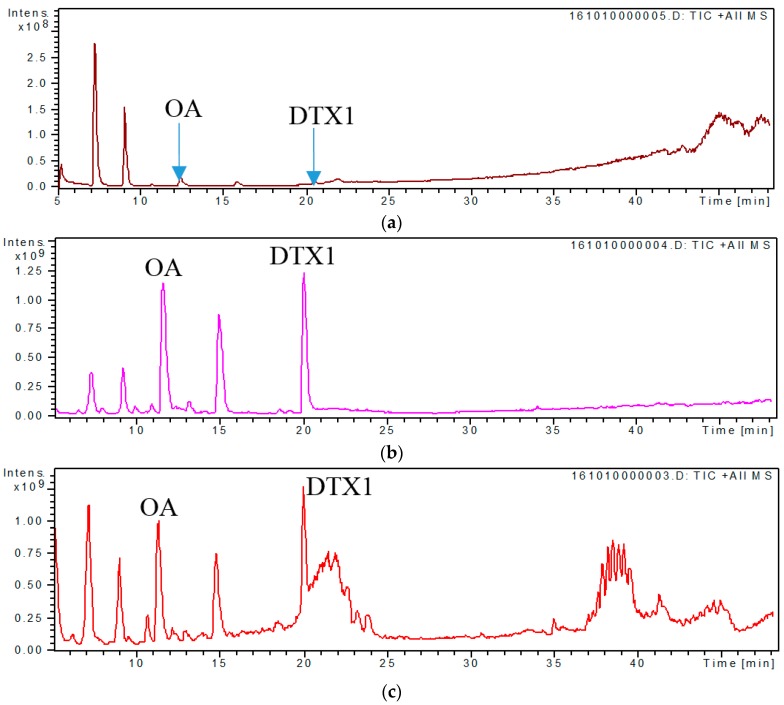
LC–MS total ion chromatograms of *P. lima* culture medium treated by three methods of (**a**) direct sampling; (**b**) solid-phase extraction; (**c**) macroporous resin passive adsorption.

**Figure 2:**
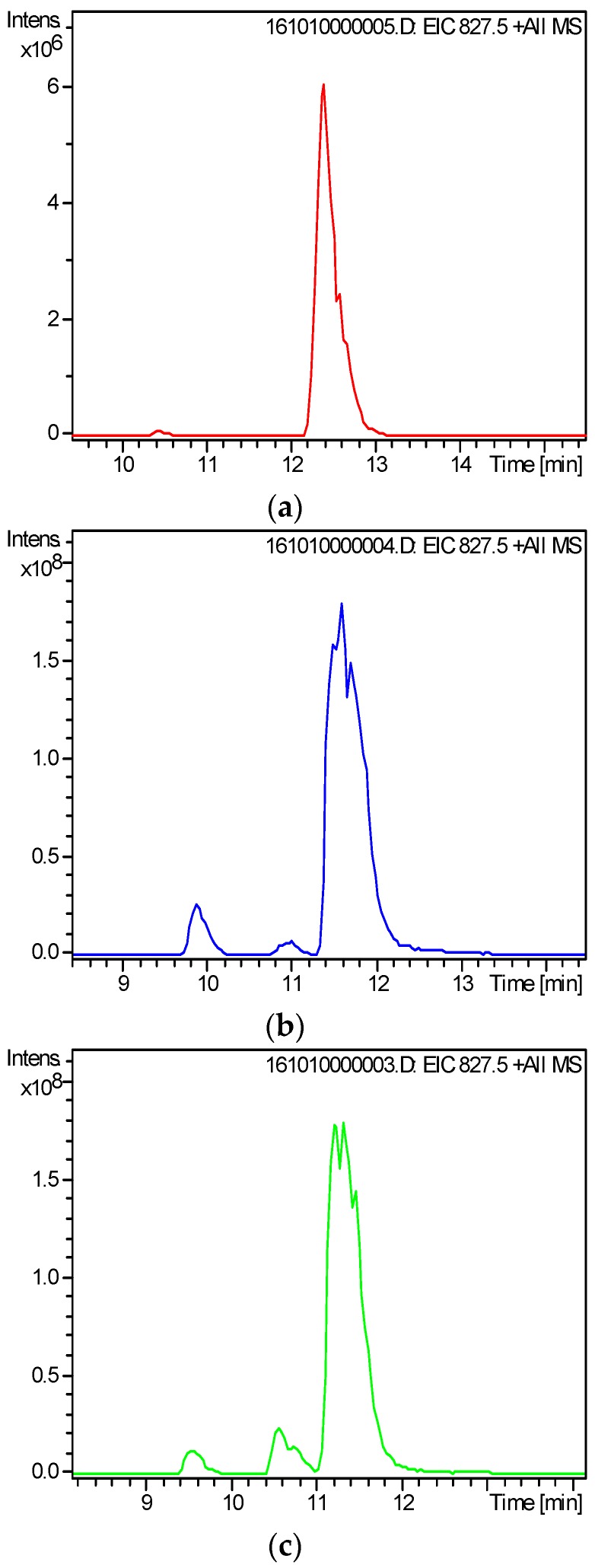
Extracted ion chromatograms of okadaic acid (OA) in *P. lima* culture medium treated by three methods of (**a**) direct sampling; (**b**) solid-phase extraction; (**c**) macroporous resin passive adsorption.

Developing a valid sample pretreatment method for the simultaneous analysis of all DSP compounds in the culture medium of marine toxigenic algae is critical. The applicability of three pretreatment methods, namely, direct sampling, SPE, and MRPA, was compared based on relevant reports [26,27,29,33,34,35]. Two typical DSP toxins, OA and DTX1 (Figure 1), were detected in the test solution obtained through these pretreatment methods, which indicated that these three methods can be used to characterize OA and DTX1 in the culture medium of toxigenic algae. However, the peak intensities and signal-to-noise ratio (S/N) of OA and DTX1 in Figure 1b,c are apparently higher than those in Figure 1a, which imply that SPE and MRPA can improve the detection sensitivity of extracellular DSP compounds in the culture medium. The extracted ion chromatograms (EICs) of OA at *m*/*z* 827.5 [M + Na]^+^ described in Figure 2b,c show distinct isomeric peaks of OA, whereas those in Figure 2a are unclear. These findings further demonstrate that the detection efficiency for low-concentration DSP compounds in the culture medium can be increased by SPE or MRPA. Compared with MRPA (Figure 1c), SPE (Figure 1b) has a better cleanup for the characterization of extracellular DSP compounds by LC–MS.

**Figure 3:**
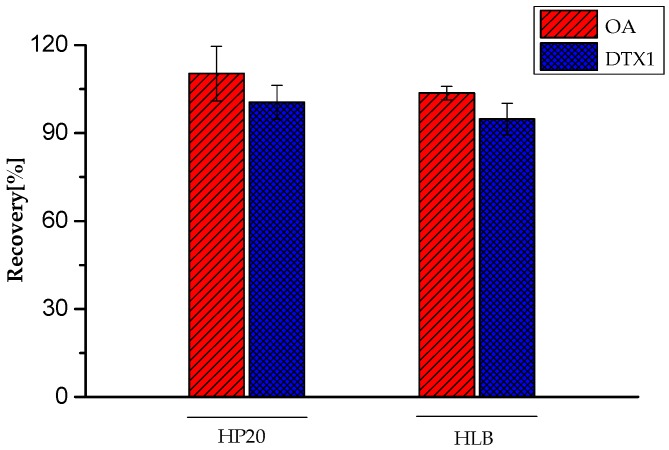
Recovery of OA, dinophysistoxin-1 (DTX1) treated by solid phase extraction (HLB) and macroporous resin passive adsorption (HP20). Error bars of triplicate analysis are included.

Furthermore, the applicability of SPE and MRPA for the quantitative analysis of extracellular DSP toxins was investigated. As shown in Figure 3, the recoveries of OA and DTX1 for SPE were 103.6% and 94.8%, respectively, with RSD (*n* = 3) ≤ 5.4%. For MRPA, the recoveries of OA and DTX1 were 110.3% and 100.6%, respectively, with RSD (*n* = 3) ≤ 9.3%.

The results above indicate that direct sampling can be used as a pretreatment method for the screening and identification of high-concentration extracellular DSP toxins in the culture medium of toxigenic algae. This method has several advantages, such as simplicity, no consumption of organic solvents, and low cost. In addition, direct sampling allows the identification of main extracellular DSP toxins in the culture medium through LC–MS even when the culture medium volume is very small (only a few milliliters or tens of milliliters). Aside from the main DSP toxins, low-content extracellular DSP compounds can also be screened and identified in the culture medium through SPE and MRPA. Compared with MRPA, SPE requires much lesser time and organic solvents and can obtain a better cleanup effect for the enrichment of the extracellular DSP compounds in the culture medium. Therefore, SPE was selected as the preferred sample pretreatment method for the profiling and identification of extracellular DSP compounds in the culture medium of harmful marine algae.

### 2.2. Method Performance

Results from the instrumental precision of LC–HR–MS for two typical DSP toxins, OA and DTX1, are summarized in Table S1 (Supplementary Materials). The RSDs of peak area and retention time were ≤ 2.61% and ≤ 0.31%, respectively. The mass error of the measured exact mass for both OA and DTX1 was ≤ 5 ppm, which indicated satisfactory results in terms of precision and the exact mass of the method. The limits of detection (LODs) of OA and DTX1 were 12 and 25 pg, respectively. In general, sensitivity was able to completely meet the requirement for the screening and identification of extracellular DSP compounds in the real culture medium of harmful marine algae.

Matrix effects (ion suppression or ion enhancement) may affect the quantitative accuracy for LC–MS analysis, especially when electrospray ionization (ESI) source is used [36]. Thus, the matrix effect on the LC–MS/MS for DSP toxin quantification was determined. Ion suppression or ion enhancement of OA (+15.06%) and DTX1 (−13.35%) was observed (Table 1), indicating that the matrix effect cannot be ignored. Therefore, matrix-matched calibration standard curves were used for the quantification of the extracellular DSP compounds in the culture medium to ensure the accuracy of the quantitative results.

The LC–MS/MS method was validated, and the results for precision, linearity, regression equation, correlation coefficient, LOD, and limit of quantification (LOQ) are shown in Tables S2 and S3. Good precision was obtained for OA and DTX1, with RSDs for peak areas and retention time of less than 4.20% and 1.16%, respectively. The matrix-matched calibration standard curves of target toxins showed good linear relationships with the coefficients of determination *R*^2^ ≥ 0.9990, and the LODs and LOQs of this established method were within 0.22–0.47 and 0.56–0.93 pg/mL, respectively. The results indicate that the proposed method has a considerable sensitivity and quantitative linearity, and meets the requirements for the quantitative or semi-quantitative detection of extracellular DSP compounds in the real culture medium of harmful marine algae.

### 2.3. Screening and Identification of Extracellular DSP Compounds in the Culture Medium of P. lima

**Figure 4:**
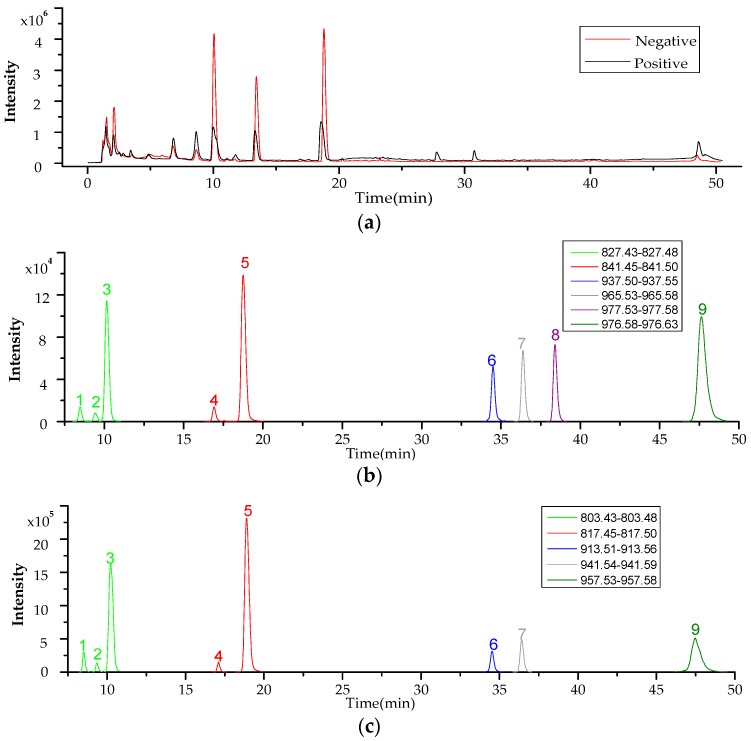
LC–HR-MS TICs and EICs of suspected DSP compounds in *P. lima* culture medium (**a**) total ion chromatograms (TICs) in both positive and negative mode of the electrospray ionization (ESI^+^ and ESI^−^); (**b**) extracted ion chromatograms(EICs) in ESI^+^; (**c**) EICs in ESI^−^.

**Figure 5:**
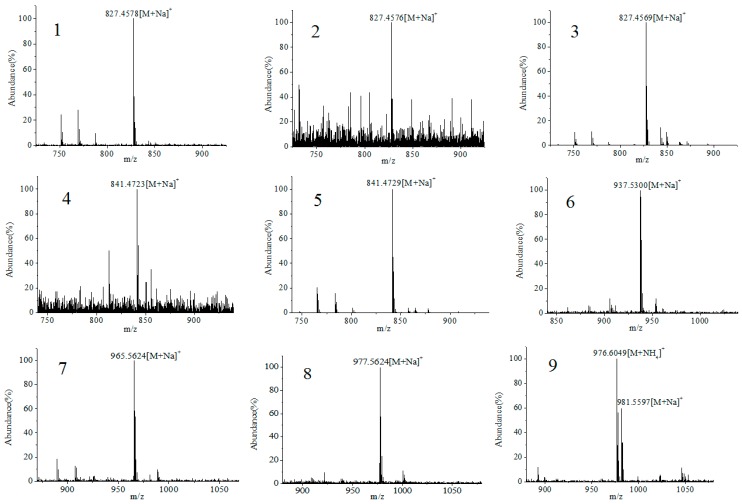
HR-MS spectra of nine suspected DSP compounds in *P. lima* culture medium.

The testing solution of *P. lima* culture medium was analyzed using the developed LC–HR–MS method in both positive and negative ion modes to obtain the crude total ion chromatogram (TIC) data (Figure 4a). Then, the exact mass data of all 93 known DSP compounds (Table S4) were entered to extract MS signals from the LC–HR–MS TICs. As shown in Figure 4b,c, several suspected EIC peaks were obtained through signal extraction, whether in positive or negative ion mode. The HR–MS spectra of each suspected peak are provided in Figure 5. The process shown below exemplifies the identification procedure for typical peaks by LC–HR–MS and LC–MS/MS.

**Figure 6:**
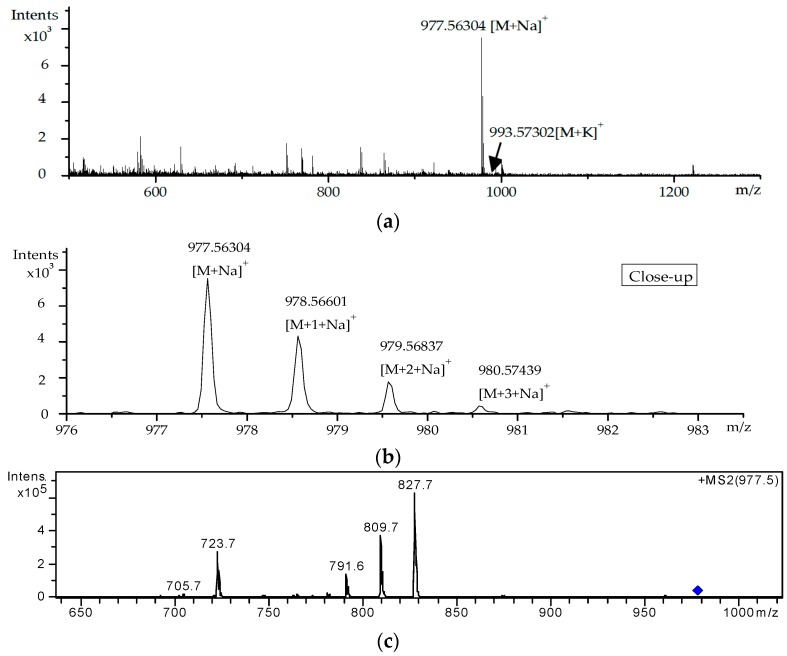
(**a**) HR-MS spectra of suspected OA-D10a/b; (**b**) Close-up of the HR-MS spectra in (**a**); (**c**) MS/MS spectra of suspected OA-D10a/b.

For the DSP toxins without a commercially available reference standard, peak 8 was taken as an example to introduce the specific process for screening and identification. The 8th peak corresponds to *m*/*z* 977.5630 obtained by accurate mass extraction (extraction window of *m*/*z* 977.53–977.58). The MS spectrum close-up of this peak is provided in Figure 6b. The possible molecular formula of this compound was deduced as C_54_H_82_O_14_ using Masshunter software, which was consistent with the chemical composition of OA-D10a/b. As shown in Table 2, a relative mass error of −3.53 ppm was encountered for this compound. Moreover, the measured isotopic value clearly matched the theoretically calculated value (Figure 6b). Thus, the molecular formula of this compound was confirmed as C_54_H_82_O_14_, whereby the compound was tentatively identified as OA-D10a/b. The above findings indicate that HR–MS combined with accurate mass information can meet the requirements for the rapid screening and preliminary identification of DSP compounds in the culture medium.

**Figure 7:**
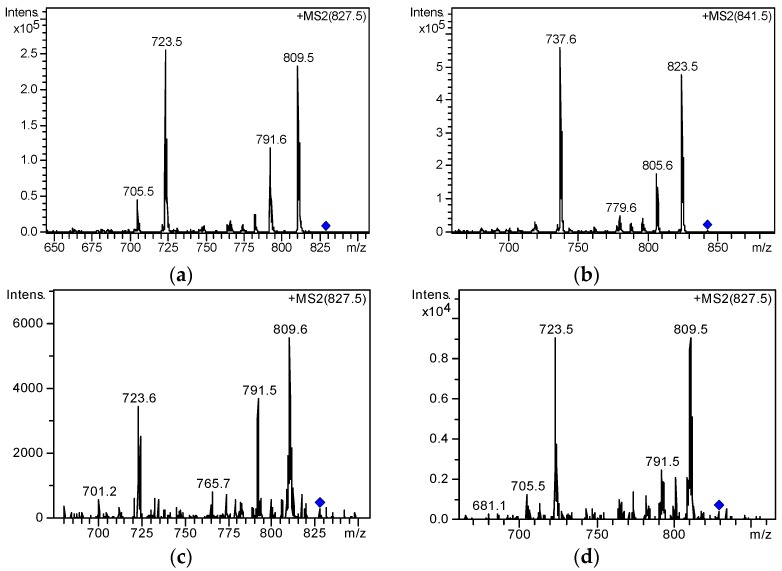

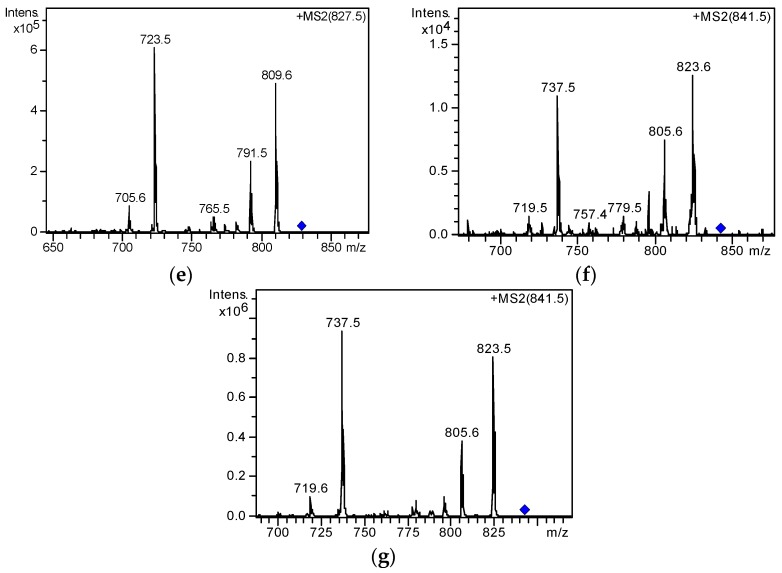
MS/MS spectra of DSP compounds in *P. lima* culture medium and in mixed standard solution; (**a**,**b**) MS/MS spectra of OA, DTX1 in mixed standard solution; (**c**–**g**) MS/MS spectra of peak 1–5 from *P. lima* culture medium.

LC–MS/MS was employed to further verify the compound. Figure 6c shows the MS/MS spectrum of the suspected OA-D10a/b. Compared with the MS/MS spectrum of OA standard (Figure 7), a typical fragment ion (*m*/*z* 827.7) of peak 8 was consistent with the quasi-molecular ion ([M + Na]^+^) of OA. This result indicates that peak 8 is a member of the OA family. Other fragment ions including *m*/*z* 809.6, 791.5, 723.5, and 705.5 in Figure 6c were in agreement with the fragment ions of OA. Thus, the compound was ultimately confirmed as OA-D10a/b. Li et al. [18] also characterized OA-D10a/b by LC–MS/MS and obtained different MS fragmentation information, which might be due to the different types of mass spectrometer used.

**Figure 8:**
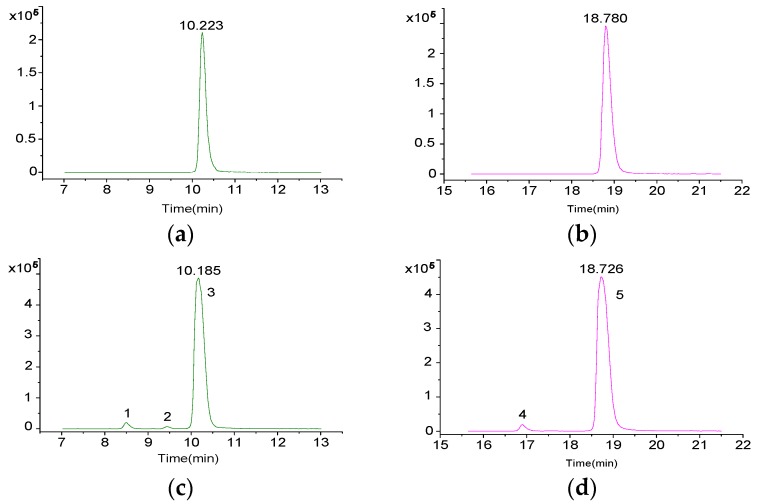
EICs of OA, DTX1 in *P. lima* culture medium and in mixed standard solution (**a**) EIC of OA in mixed standard solution; (**b**) EIC of DTX1 in mixed standard solution; (**c**) EIC of OA in *P. lima* culture medium; (**d**) EIC of DTX1 in *P. lima* culture medium.

For the DSP toxins with commercially available reference standards, retention time and fragmentation information were applied as evidence for verification by comparing with the values of the reference standard. The process described below exemplifies the confirmation of peaks 1, 2, 3, 4, and 5, as shown in Figure 4b. The MS spectra (Figure 5) of peaks 1, 2, and 3 contained the base peak of *m*/*z* 827.45, suggesting that the three compounds are OA suspects. Similarly, the exact molecular weights of peaks 4 and 5 were consistent with that of DTX1. However, we could not determine the peaks of OA and DTX1. Thus, LC–MS/MS was applied to verify the suspected toxins. By comparing with the retention times of standard OA and DTX1, peaks 3 and 5 were able to be preliminarily identified as OA and DTX1 (Figure 8), respectively. Subsequently, the fragment ions of peak 3 and OA, and peak 5 and DTX1 were found to be coincident by comparing the MS/MS spectra in Figure 7. As a consequence, peaks 3 and 5 (Figure 7c,e) were ultimately determined to be OA and DTX1, respectively.

In addition, MS/MS analysis was performed on peaks 1, 2, and 4 (Figure 7c,d,f). The characteristic fragment ions of peaks 1, 2 and peak 4 were in agreement with the OA and DTX1 reference standards, respectively. This finding indicates that peaks 1, 2 and peak 4 are the isomeric peaks of OA and DTX1, respectively. Due to the absence of reference standards, the isomers of OA and DTX1 can only be determined by combining with the relevant literature [20,31,37]. Peaks 1 (Figure 7c) and 2 (Figure 7d) may be one or both of 19-*epi*-OA, DTX2, DTX2b, or DTX2c. Although peak 4, OA methyl ester, and 35S DTX1 are isomers, the characteristic fragment ions of peak 4 (Figure 7f) were consistent with that of DTX1. Therefore, peak 4 was speculated to be 35S DTX1 instead of OA methyl ester.

The screening and identification of other DSP compounds in the culture medium of *P. lima* was similar to the procedure described above. In this study, nine extracellular DSP compounds were successfully identified in the culture medium of *P. lima*. The retention time, molecular formula, detection ion, and mass deviation of all DSP compounds are listed in Table 2. The relative deviation between the measured and calculated values of the accurate molecular weight of each compound was smaller than 6 ppm, which was in line with the accuracy requirements for screening by HR–MS. In addition, the MS/MS characteristics of all DSP compounds were similar because of their similar molecular structures. During MS analysis, the DSP compounds became prone to [M + Na]^+^ ion, with higher abundance in ESI positive mode, and were accompanied by [M + K]^+^ ion in trace amounts. In ESI negative mode, [M − H]^−^ ion was easily generated. However, all DSP compounds had lower sensitivity in negative mode except for OA and DTX1, as well as their isomers. During MS/MS analysis, the main fragment ions were *m*/*z* 809, 791, 723, and 705 for the compounds of the OA group and *m*/*z* 823, 805, 737, and 719 for the compounds of the DTX1 group, which were produced by a series of losses of H_2_O, H_2_O, HOCONa, and H_2_O from the precursor [M+Na]^+^ ion. As mentioned in the literature [4,7,11,13], only OA and DTX1 were identified in the culture medium of *P. lima*. In this study, the profile features of DSP compounds in the culture medium of *P. lima* were elucidated by LC–HR–MS and LC–MS/MS, laying a good foundation for research on extracellular DSP toxins released by marine toxigenic algae.

### 2.4. Contents of Extracellular DSP Compounds in the Culture Medium of P. lima at Different Growth Stages

Present research on the excretion of DSP toxins for marine toxigenic algae is limited [2,5,40]. In the present study, extracellular DSP compounds in the culture medium collected 8, 16, 22, and 25 days after the inoculation of *P. lima* were identified and quantified. Different kinds of DSP compounds are collected from the culture medium of *P. lima* at different incubation times (Table S5). When the incubation time was 8 days, nine DSP compounds were detected in the algal cells and the culture medium of *P. lima*. When the incubation time periods were 16, 22, and 25 days, eight DSP toxins were detected intracellularly. Furthermore, only 5, 7, and 6 DSP toxins, respectively, were detected in the culture medium.

**Figure 9:**
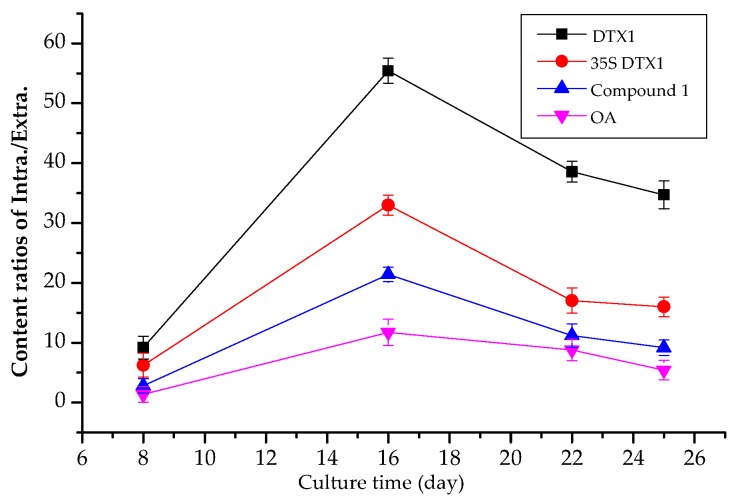
The ratios of intracellular to extracellular content of four major DSP toxins under different growth period of *P. lima*.

Qantitative/semi-quantitative results of extracellular DSP compounds in the culture medium of *P. lima* are shown in Table 3. The content of each intercellular DSP compound increased gradually as the culture time was prolonged (e.g., OA content increased from 2.26 pg/cell to 14.45 pg/cell, and DTX1 content increased from 19.56 pg/cell to 66.25 pg/cell). Meanwhile, the content of extracellular DSP compounds initially decreased and then increased, which agreed with the research results of Nascimento et al. [16]. In the initial stage of culture (8 days), nutrients in the culture medium were abundant, and the number of cells increased rapidly due to the rapid division in the exponential growth phase, which may have triggered the reduction in the content of extracellular toxins per unit cell. At the same period, the total amount of extracellular DSP compounds in the culture medium was also reduced by 2.43%–45.69%, which can probably be attributed to the reabsorption and reuse of DSP compounds at the rapid growth stage. When the rate of cell division slowed down, aging cultures and apoptosis appeared to promote a passive release of DSP compounds. Thus, the total content of extracellular DSP compounds increased significantly [2], whereas the accumulation rate of intracellular toxins slowed down. The ratios of intracellular to extracellular contents of major DSP toxins depicted in Figure 9 initially increased, and then decreased, which was consistent with the explanation above. As shown in Table 3, the content of intercellular DTX1 at the different growth stages of *P. lima* was significantly higher (about 3–8 times) than that of OA. However, the contents of extracellular DTX1 and OA showed no significant difference. This finding might be the result of a very fast enzymatic hydrolysis into OA diol ester derivatives from OA sulfated diester (the initial form intracellularly), which was further hydrolyzed at a low rate to yield OA [27].

The total content of DSP compounds per unit cell (intracellular and extracellular) increased continuously, suggesting that the production rate of DSP compounds was greater than the elimination rate throughout incubation. During the 25-day incubation period, the content of intracellular DSP compounds was essentially higher than that in the culture medium. However, the content of 5,7-dihydroxy-2,4-dimethylene-heptyl okadaate was the opposite, which might be caused by its different metabolic patterns, relative to other DSP compounds. The total concentration of extracellular DSP compounds in the culture medium was within 57.70–79.63 μg/L, and accounted for 4.81%–16.31% of the total toxin content. Hence, the content of extracellular DSP compounds in the culture medium should not to be underestimated. Li et al. [26] examined the monthly variations of DSP toxins in the coastal seawaters of Qingdao, with an OA concentration range of 1.41–89.52 ng/L. Nevertheless, the concentration ranges of OA (19.9–34.0 μg/L) and DTX1 (15.2–27.9 μg/L) in the culture medium of *P. lima* were much higher than those in seawater. Previous studies [41,42] reported that the algal cell density is generally within 10^2^–10^6^ cells/mL during red tide outbreaks. Similarly, the cell density in this work was within the 1.28–2.58 × 10^4^ cells/mL during *P. lima* incubation, which corresponded to the density at the time of red tide. Although the reality in seawater and in vitro culture media is indeed different in terms of aspects such as nutrients, temperature, light and the stimulation of harsh environment, all of which will likely affect the production of toxins. The concentration of DSP compounds in vitro culture media can still provide a certain reference for assessing the environmental risk of extracellular toxins during red tide outbreaks. It can be deduced that the concentrations of DSP toxins could potentially reach tens of or even hundreds of micrograms per liter in the seawater during red-tide outbreaks of DSP-producing algae. If so, extracellular toxins would pose a huge threat to various marine life, especially to cultivated shellfish.

### 2.5. Screening of Extracellular DSP Compounds in the Culture Medium of P. minimum

It has been pointed out that *P. minimum* is one of the producers of DSP toxins [8,43], but specific case reports for the detection of DSP compounds in this type of algae and culture medium are still lacking. Intercellular and extracellular DSP compounds were screened in *P. minimum* cultured in the laboratory with different culture times. Results displayed that intracellular and extracellular DSP compounds were undetected. In summary, *P. minimum* from the Shenzhen Bay of China is incapable of producing DSP toxins when cultured in the laboratory.

## 3. Conclusions

The proposed method based on LC–HR–MS and LC–MS/MS for the profiling of extracellular DSP compounds is effective for the rapid identification and quantification of extracellular compounds associated with DSP in the culture medium of harmful marine algae. This method can be used to determine differences between the intracellular and extracellular DSP compounds of marine toxigenic algae. The developed method might provide a basis for further research on the characterization, environmental behavior, and toxicity assessment of extracellular DSP compounds from different harmful marine algae. Nine extracellular DSP compounds were successfully identified in the culture medium of *P. lima*, of which seven DSP compounds were detected for the first time. The total contents of extracellular DSP compounds in the culture medium of *P. lima* were within 57.70–79.63 μg/L, which indicated that the extracellular DSP compounds released by *P. lima* could not be neglected. Future studies should focus on the release kinetics of extracellular DSP compounds and the effect of environmental factors on DSP-producing algae.

## 4. Materials and Methods

### 4.1. Materials and Reagents

Ultrapure water was produced by Milli-Q Water Purification System (Millipore Bedford, Billerica, MA, USA). Methanol and acetonitrile (HPLC grade) were purchased from Merck (Darmstadt, Germany). MS-grade ammonium hydroxide (≥ 25%) was obtained from Fluka (St. Louis, MO, USA). Oasis HLB cartridges (200 mg, 6 mL) were purchased from Waters (Medford, OR, USA). HP20 macroporous adsorption resins were purchased from Mitsubishi Chemical Corporation (Tokyo, Japan). The reference standards of OA and DTX1 were purchased from Express Technology Co., Ltd. (Beijing, China).

### 4.2. Preparation of Standard Solutions

Stock standard solutions of OA and DTX1 were prepared by dissolving and diluting the reference standards in methanol. Mixed stock solutions were prepared by diluting stock standard solutions in methanol at certain proportions. The mixed standard solution consisted of 15 μg/L OA and 25 μg/L DTX1. All standard solutions were stored at −20 °C.

### 4.3. Microalgae Cultivation and Collection

*P. lima* (GY-H57) and *P. minimum* (GY-H38) strains were obtained from Shanghai Guangyu Biological Technology Co., Ltd. (Shanghai, China). Algae in the exponential growth period were inoculated into *f*/2 culture medium [44] with a salinity of 30‰. The seawater used in the preparation of the culture medium was subjected to sterilization at high temperature (121 ± 2 °C). The algae were grown at 20 ± 1.0 °C in a temperature-controlled light incubator at an irradiance of 3000 Lux and a light:dark cycle of 12:12 h. The number of cells was determined using a blood count plate. The algal culture was divided into four batches (in triplicate), and the incubation times were 8, 16, 22, and 25 days. The algae and culture medium were separated by high-speed freezing centrifugation at a temperature of 4 °C and a speed of 4000 r/min. The algae were freeze-dried into powder and placed in a refrigerator at −20 °C. The algae culture medium collected was stored in a refrigerator at 4 °C.

### 4.4. Sample Preparation

#### 4.4.1. Extraction of DSP Compounds from Toxigenic Algae

Extraction was performed as previously described [33]. In brief, 100.0 mg of algae powder (dry weight) and 2 mL of methanol were placed in a 10 mL polypropylene centrifuge tube and disrupted by an ultrasonic cell disruptor for 3 min and then extracted in an ultrasonic bath for 30 min. The extracts were centrifuged with a high-speed refrigerated centrifuge for 10 min at 6000 rpm, and the supernatant was transferred to a volumetric flask. The extraction was repeated once. The extracts were combined twice, dried under a nitrogen stream, and then reconstituted with 1 mL of methanol. Afterward, the extracts were filtered through a 0.22 μm membrane into an LC vial and then stored at −20 °C until further analysis.

#### 4.4.2. Direct Sampling

Exactly 1 mL of culture medium was removed directly from the culture bottle of toxigenic algae and filtered through a 0.22 μm membrane (nylon 66) into an LC vial as a test solution.

#### 4.4.3. Solid-Phase Extraction

SPE was conducted with Oasis HLB cartridges. The cartridges were activated with 3 mL of methanol and 3 mL of ultrapure water. Then, 200 mL of the algae culture medium filtered through 0.45 μm glass microfiber was loaded into each cartridge at ca 1 mL/min flow rate. Subsequently, the cartridge was rinsed with 3 mL methanol/water (15:85, *v*/*v*) and then vacuum-dried for approximately 5 min. Then, the extracts were achieved by eluting the cartridges thrice with 3 mL of ammonium hydroxide/methanol (1:99, *v*/*v*). Finally, the eluate was combined and dried under a nitrogen stream, reconstituted with 1 mL of methanol, and then filtered through a 0.22 μm membrane into an LC vial for analysis.

#### 4.4.4. Macroporous Resin Passive Adsorption

HP20 resin (2.0 g) was weighed and loaded into a sachet sewed in bolting silk. The resin was activated by immersing in 95% ethanol aqueous solution for 24 h and was then gently rinsed with 95% ethanol aqueous solution and ultrapure water to remove impurities. Subsequently, the sachet was immersed in 200 mL of algae culture medium for 24 h. After 24 h of exposure, the HP20 resin was removed from the sachet, loaded into the SPE cartridge, and then rinsed with 2 mL of ultrapure water. Then, vacuum was applied to remove remaining water, and the resin was eluted with 3 mL of acetone dropwise. The collected eluent was evaporated to dryness under a nitrogen stream. The dry residue was finally reconstituted with 1 mL of methanol and filtered through a 0.22 μm membrane into an LC vial.

### 4.5. Instrumental Conditions

Chromatographic separation was performed using an Agilent 1200 Series HPLC system (Agilent Technologies, Wilmington, DE, USA) consisting of a vacuum degasser, a quaternary pump, an autosampler, and a ZORBAX Extend-C18 analytical column (3 mm × 150 mm, 3.5 μm). Mobile phase A consisted of water, and mobile phase B consisted of acetonitrile/water (9:1, *v*/*v*). Both phases contained 3.3 mM ammonium hydroxide. A flow rate of 0.4 mL/min was applied. Analyses were performed by running a linear gradient starting with 20% B, followed by 30% B within 15 min, 47.5% B in 20 min, 100% B in 45 min, and finally returning to the initial condition of 20% B within 50 min. The equilibration time before the next injection was 8 min, and the injection volume was 10 μL. The column oven was maintained at 22 ± 2 °C.

DSP compounds were screened using a G1969A TOF mass spectrometer (Agilent Technologies, Wilmington, DE, USA) and 6320 series ion-trap mass spectrometer (Agilent Technologies, Wilmington, DE, USA) equipped with an ESI source. Instrument parameters were both set as follows: scan range, 400–1300 *m*/*z*; capillary voltage, 4500 V; nebulizer pressure (N_2_), 40 psi; drying gas (N_2_) flow, 11 L/min; and gas temperature, 350 °C. The other parameters of TOF/MS were as follows: fragmentor, 150 V; and skimmer voltage, 60 V. Samples were scanned in negative and positive modes. The parameters of ion-trap MS included a target mass of 810 *m*/*z*. Qualitative and quantitative detection for DSP compounds was conducted under multiple reaction monitoring (MRM) mode by using the positive ESI mode, and the optimized MS/MS parameters for DSP compounds are shown in Table 4.

### 4.6. Data Analysis

#### 4.6.1. Screening of Extracellular DSP Compounds

This study summarized all the information about DSP toxins and their derivatives (a total of 93 compounds) reported in the literature, including the molecular formulae of the compounds and the adduction peaks generated by MS analysis under negative and positive modes. For the screening of DSP compounds, the exact mass information of various additional ions was obtained by calculating the *m*/*z* values of the ion peaks [M + H]^+^, [M + NH_4_]^+^, [M + Na]^+^, [M + K]^+^, and [M − H]^−^ of each compound. The details are shown in Table S4. Peak finding was performed using Masshunter software (A02.02, Agilent Technologies, Wilmington, DE, USA) by extracting the exact mass of the expected ion with a mass window of ±25 mDa from the HR–MS full-scan TIC data. The extracted ion peaks with S/N greater than 5 were identified as valid peaks, and qualitative identification was required to determine their identities.

#### 4.6.2. Identification of Extracellular DSP Compounds

For the suspected DSP compounds, Masshunter software was used to speculate their molecular formulas (the mass deviation of the mass was set within 10 ppm), and the nitrogen rule and Masshunter software isotope matching were used to evaluate the molecular formulas. A comprehensive score of Masshunter software was used to determine whether the suspect is a DSP compound. For unequivocal confirmation, the samples with positive findings were retested by a targeted LC–MS/MS approach. For the DSP compounds without standards, the MS/MS spectra obtained by MS/MS were compared with that in the literature. For compounds with standards, the MS/MS spectra and retention times were compared with those of the standards. The allowed retention time deviation between the suspect and the standard was ±0.20 min.

#### 4.6.3. Quantification and Semi-quantification of Extracellular DSP Compounds

The quantification of extracellular OA and DTX1 in the culture medium of *P. lima* was carried out using the matrix-matched calibration standard curves. The EIC peak area of the target compound was calculated under the MRM mode and then substituted into the linear equation to calculate the sample concentration. The response value of the analyte in the sample solution should be within the linear range. For the DSP compounds without reference standards, the OA reference standard was used as a reference. In addition, semi-quantification was carried out in the same way as described above.

## Figures and Tables

**Table 1:** Effects of sample matrix on the determination of OA and DTX1 for LC-MS/MS analysis (*n* = 3).

Toxins	Peak Area (Matrix)	Relative Standard Deviation (RSD%)	Peak Area (Methanol)	(RSD%)	Signal Suppression (%)
OA	11,759,724	3.32	10,220,561	1.14	+15.06
DTX1	13,774,916	2.98	15,896,689	2.16	−13.35

**Table 2:** The exact mass and identification of DSP compounds in *P. lima* culture medium.

Peak	Retention Time (min)	Detected Ion	Measured (*m/z*)	Theoretial (*m*/*z*)	Error (ppm)	Chemical Fomula	Toxin ID	References
1	8.5	[M + Na]^+^	827.4576	827.4552	−2.97	C_44_H_68_O_13_	19-epi-OA /DTX2/DTX2b/DTX2c	[31,37]
2	9.4	[M + Na]^+^	827.4578	827.4552	−3.22	C_44_H_68_O_13_		
3	10.2	[M + Na]^+^	827.4569	827.4552	−2.1	C_44_H_68_O_13_	OA	[20,31,37]
4	16.9	[M + Na]^+^	841.4723	841.4709	−1.76	C_45_H_70_O_13_	35S DTX1	[31]
5	18.8	[M + Na]^+^	841.4729	841.4709	−2.49	C_45_H_70_O_13_	DTX1	[20,31,37]
6	34.6	[M + Na]^+^	937.5300	937.5284	−1.77	C_51_H_78_O_14_	OA-D7b	[31,38]
7	36.4	[M + Na]^+^	965.5624	965.5597	−2.89	C_53_H_82_O_14_	OA-D9b	[31,38,39]
8	38.6	[M + Na]^+^	977.5624	977.5597	−3.53	C_54_H_82_O_14_	OA-D10a/b	[18,31]
9	48.6	[M + NH_4_]^+^	976.6049	976.5992	−5.86	C_53_H_82_O_15_	5,7-dihydroxy-2,4-dimethylene-heptyl okadaate	[20,31,39]

**Table 3:** Quantitative results of DSP compounds in the culture medium under different incubation times of *P. lima*.

Incubation Time (Days)		8	16	22	25
**Algae density (×10^4^ cells/mL)**		1.31	2.58	1.78	1.28
**OA (pg/cell)**	**Intracellular**	2.26	9.62	9.85	14.45
	**Extracellular**	1.65	0.82	1.12	2.66
	**Total**	3.91	10.44	10.97	17.11
	**Intracellular/Extracellular**	1.37	11.73	8.79	5.43
**Extracellular concentration (μg/L)**		21.63	21.10	19.93	34
**Compound 1 (pg/cell)**	**Intracellular**	5.78 × 10^−2^	0.20	0.17	0.32
	**Extracellular**	2.05 × 10^−2^	9.34 × 10^−3^	1.52 × 10^−2^	3.49 × 10^−2^
	**Total**	7.83 × 10^−2^	0.21	0.19	0.35
	**Intracellular/Extracellular**	2.82	21.41	11.18	9.17
**Extracellular concentration (μg/L)**		0.27	0.24	0.27	0.46
**35S DTX1 (pg/cell)**	**Intracellular**	2.14 × 10^−2^	5.21 × 10^−2^	7.84 × 10^−2^	0.14
	**Extracellular**	3.43 × 10^−3^	1.58 × 10^−3^	4.60 × 10^−3^	8.58 × 10^−3^
	**Total**	2.48 × 10^−2^	6.92 × 10^−2^	8.30 × 10^−2^	0.15
	**Intracellular/Extracellular**	6.24	32.97	17.04	15.99
**Extracellular concentration (μg/L)**		4.49 × 10^−2^	4.07 × 10^−2^	8.17 × 10^−2^	0.11
**DTX1 (pg/cell)**	**Intracellular**	19.56	32.70	50.91	66.25
	**Extracellular**	2.13	0.59	1.32	1.91
	**Total**	21.69	33.29	52.23	68.16
	**Intracellular/Extracellular**	9.18	55.42	38.57	34.69
**Extracellular concentration (μg/L)**		27.90	15.15	23.55	24.35
**OA-D7b (pg/cell)**	**Intracellular**	0.09	0.67	1.01	0.68
	**Extracellular**	3.54 × 10^−3^	—	—	—
**Extracellular concentration (μg/L)**		4.64 × 10^−2^	—	—	—
**OA-D9b (pg/cell)**	**Intracellular**	3.66	7.54	14.57	16.93
	**Extracellular**	1.91 × 10^−3^	—	—	—
**Extracellular concentration (μg/L)**		2.50 × 10^−2^	—	—	—
**5,7-dihydroxy-2,4-dimethylene-heptyl okadaate (pg/cell)**	**Intracellular**	0.105	0.15	0.18	—
	**Extracellular**	0.594	1.17	1.53	1.62
**Extracellular concentration (μg/L)**		7.78	30.13	27.196	20.695
**OA-D10a/b (pg/cell)**	**Intracellular**	1.25	2.93	5.41	6.23
	**Extracellular**	1.1 × 10^−3^	—	—	—
**Extracellular concentration (μg/L)**		1.44 × 10^−2^	—	—	—
**Total extracellular concentration (μg/L)**		57.70	66.72	70.92	79.63

—: No limit of quantitation reached.

**Table 4:** MS/MS parameters for MRM acquisition windows in the detection of DSP compounds.

Toxins	Retention Time (min)	Chemical Formula	Precursor Ion (*m*/*z*)	Product Ions (*m*/*z*)	Collision Energy Ampl/V
OA	10.2	C_44_H_68_O_13_	[M + Na]^+^ (827.5)	809.6/723.6	1.0
DTX1	18.8	C_47_H_70_O_14_	[M + Na]^+^ (841.5)	737.6/823.6	1.5
19-epi-OA/DTX2/DTX2b/DTX2c	8.5	C_44_H_68_O_13_	[M + Na]^+^ (827.5)	809.6/723.6	1.0
	9.4	C_44_H_68_O_13_	[M + Na]^+^ (827.5)	809.6/723.6	1.0
35S DTX1	16.9	C_47_H_70_O_14_	[M + Na]^+^ (841.5)	737.6/823.6	1.5
OA-D7b	34.6	C_51_H_78_O_14_	[M + Na]^+^ (937.5)	827.7/809.6	1.5
OA-D9b	36.4	C_53_H_82_O_14_	[M + Na]^+^ (965.6)	827.7/809.6	1.5
OA-D10a/b	38.6	C_54_H_82_O_14_	[M + Na]^+^ (977.6)	827.7/809.6	1.5
5,7-dihydroxy-2,4-dimethylene-heptyl okadaate	48.6	C_53_H_82_O_15_	[M + NH_4_]^+^ (976.6)	822.7/804.6	1.5

## Supplementary Materials

The following are available online at www.mdpi.com/2072-6651/9/10/308/s1, Table S1: Precision of HR-MS instrument, Table S2: Precision of Trap/MS instrument, Table S3: Results of linearity and sensitivity for OA and DTX1, Table S4: Formula and its theoretical precise molecular mass of 93 DSP compounds, Table S5: Detection of DSP compounds at different culture times of *P. lima*, Detailed experimental process of the evaluation of matrix effect and the validation of the method.

## References

[1] Granéli E., Turner J.T. (2007). Ecology of harmful algae. Eos Trans. Am. Geophys. Union.

[2] Smith J.L., Tong M., Fux E., Anderson D.M. (2012). Toxin production, retention, and extracellular release by *Dinophysis* acuminate during extended stationary phase and culture decline. Harmful Algae.

[3] Trainer V.L., Moore L., Bill B.D., Adams N.G., Harrington N., Borchert J., Silva D.A.M.D., Eberhart B.T.L. (2013). Diarrhetic shellfish toxins and Oother lipophilic toxins of human health concern in washington state. Mar. Drugs.

[4] Taylor M., McIntyre L., Ritson M., Stone J., Bronson R., Bitzikos O., Rourke W., Galanis E. (2013). Outbreak of diarrhetic shellfish poisoning associated with mussels, British Columbia, Canada. Mar. Drugs.

[5] Nielsen L.T., Krock B., Hansen P.J. (2013). Production and excretion of okadaic acid, pectenotoxin-2 and a novel dinophysistoxin from the DSP-causing marine dinoflagellate *Dinophysis* acuta–Effects of light, food availability and growth phase. Harmful Algae.

[6] Suzuki T., Baba M.K., Sugawara R., Kamiyama T. (2009). LC-MS/MS analysis of okadaic acid analogues and other lipophilic toxins in single-cell isolates of several *Dinophysis* species collected in hokkaido, Japan. Harmful Algae.

[7] Hu T., LeBlanc P., Burton I.W., Walter J.A., McCarron P., Melanson J.E., Strangman W.K., Wright J.L.C. (2017). Sulfated diesters of okadaic acid and DTX-1: Self-protective precursors of diarrhetic shellfish poisoning (DSP) toxins. Harmful Algae.

[8] Zhao X.F., Ji R. (2006). Advance in the research of diarrhetic shellfish poisons. China Trop. Med..

[9] Lee T.C.H., Fong F.L.Y., Ho K.C., Lee F.W.F. (2016). The mechanism of diarrhetic shellfish poisoning toxin production in *Prorocentrum* spp.: Physiological and molecular perspectives. Toxins.

[10] Vanucci S., Guerrini F., Milandri A., Pistocchi R. (2010). Effects of different levels of N- and P-deficiency on cell yield, okadaic acid,DTX-1, protein and carbohydrate dynamics in the benthic dinoflagellate *Prorocentrum lima*. Harmful Algae.

[11] Hattenrath-Lehmann T.K., Marcoval M.A., Mittlesdorf H., Goleski J.A., Wang Z.H., Haynes B., Morton S.L., Gobler C.J. (2015). Nitrogenous nutrients promote the growth and toxicity of *Dinophysis acuminata* during Estuarine Bloom events. PLoS ONE.

[12] López-Rosales L., Gallardo-Rodríguez J.J., Sánchez-Mirón A., Cerón-García M.C., Belarbi E.H., García-Camacho F., Molina-Grima E. (2014). Simultaneous effect of temperature and irradiance on growth and okadaic acid production from the marine dinoflagellate *Prorocentrum belizeanum*. Toxins.

[13] Ajuzie C.C. (2008). Toxic *Prorocentrum lima* induces abnormal behaviour in juvenile sea bass. J. Appl. Phycol..

[14] Ajuzie C.C. (2007). Palatability and fatality of the dinoflagellate *Prorocentrum lima* to Artemia salina. J. Appl. Phycol..

[15] Vale P., Veloso V., Amorim A. (2009). Toxin composition of a *Prorocentrum lima* strain isolated from the Portuguese coast. Toxicon.

[16] Nascimento S.M., Purdie D.A., Morris S. (2005). Morphology, toxin composition and pigment content of *Prorocentrum lima* strains isolated from a coastal lagoon in southern UK. Toxicon.

[17] Gerssen A., Mulder P.P., McElhinney M.A., Boer J.D. (2009). Liquid chromatography–tandem mass spectrometry method for the detection of marine lipophilic toxins under alkaline conditions. J. Chromatogr. A.

[18] Li J., Li M., Pan J., Liang J., Zhou Y., Wu J. (2012). Identification of the okadaic acid-based toxin profile of a marine dinoflagellate strain *Prorocentrum lima* by LC-MS/MS and NMR spectroscopic data. J. Sep. Sci..

[19] Orellana G., Bussche J.V., Meulebroek L.V., Vandegehuchte M., Janssen C., Vanhaecke L. (2014). Validation of a confirmatory method for lipophilic marine toxins in shellfish using UHPLC–HR–Orbitrap MS. Anal. Bioanal. Chem..

[20] Orellana G., Meulebroek L., Vooren S.V., Rijcke M.D., Vandegehuchte M., Janssen C.R., Vanhaecke L. (2015). Quantification and profiling of lipophilic marine toxins in microalgae by UHPLC coupled to high-resolution orbitrap mass spectrometry. Anal. Bioanal. Chem..

[21] Gerssen A., Mulder P.P., Boer J.D. (2011). Screening of lipophilic marine toxins in shellfish and algae: Development of a library using liquid chromatography coupled to orbitrap mass spectrometry. Anal. Chim. Acta.

[22] Zendong Z., McCarron P., Herrenknecht C., Sibat M., Amzil Z., Cole R.B., Hess P. (2015). High resolution mass spectrometry for quantitative analysis and untargeted screening of algal toxins in mussels and passive samplers. J. Chromatogr. A.

[23] Gerssen A., McElhinney M.A., Mulder P.P., Bire R., Hess P., Boer J.D. (2009). Solid phase extraction for removal of matrix effects in lipophilic marine toxin analysis by liquid chromatography-tandem mass spectrometry. Anal. Bioanal. Chem..

[24] McCarthy M., Pelt F.N.A.M.V., Bane V., O’Halloran J., Furey A. (2014). Application of passive (SPATT) and active sampling methods in the profiling and monitoring of marine biotoxins. Toxicon.

[25] Fan L., Sun G., Qiu J.B., Ma Q., Hess P., Li A. (2014). Effect of seawater salinity on pore-size distribution on a poly (styrene)-based HP20 resin and its adsorption of diarrhetic shellfish toxins. J. Chromatogr. A.

[26] Li X., Li Z., Chen J., Shi Q., Zhang R., Wang S., Wang X. (2014). Detection, occurrence and monthly variations of typical lipophilic marine toxins associated with diarrhetic shellfish poisoning in the coastal seawater of Qingdao City, China. Chemosphere.

[27] Fux E., Marcaillou C., Mondeguer F., Bire R., Hess P. (2008). Field and mesocosm trials on passive sampling for the study of adsorption and desorption behaviour of lipophilic toxins with a focus on OA and DTX1. Harmful Algae.

[28] Li A., Ma F., Song X., Yu R., Brissard C., Tixier C., Mondeguer F., Séchet V., Amzil Z., Hess P. (2011). Dynamic adsorption of diarrhetic shellfish poisoning (DSP) toxins in passive sampling relates to pore size distribution of aromatic adsorbent. J. Chromatogr. A.

[29] Zendong Z., Herrenknecht C., Abadie E., Brissard C., Tixier C., Mondeguer F., Séchet V., Amzil Z., Hess P. (2014). Extended evaluation of polymeric and lipophilic sorbents for passive sampling of marine toxins. Toxicon.

[30] Gao L.Y., Wang Y.L., Chen J.H., Wang S., Shi X.X., Shi H.H., Zheng L. (2016). Determination of eight typical lipophilic algae toxins in marine microalgae powder using high-performance liquid chromatography-ion trap mass spectrometry. Mar. Sci..

[31] Chen J.H., Li X., Wang S., Chen F.R., Cao W., Sun C.J., Zheng L., Wang X.R. (2017). Screening of lipophilic marine toxins in marine aquaculture environment using liquid chromatography-mass spectrometry. Chemosphere.

[32] Wang Y.L., Chen J.H., Li Z.Y., Wang S., Shi Q., Cao W., Zheng X.L., Sun C.J., Wang X.R., Zheng L. (2015). Determination of typical lipophilic marine toxins in marine sediments from three coastal bays of China using liquid chromatography–tandem mass spectrometry after accelerated solvent extraction. Mar. Pollut. Bull..

[33] Chen J.H., Wang Y.L., Pan L., Shen H.H., Fu D., Fu B.Q., Sun C.J., Zheng L. (2017). Separation and purification of two minor typical diarrhetic shellfish poisoning toxins from harmful marine microalgae via combined liquid chromatography with mass spectrometric detection. J. Sep. Sci..

[34] These A., Scholz J., Preiss-Weigert A. (2009). Sensitive method for the determination of lipophilic marine biotoxins in extracts of mussels and processed shellfish by high-performance liquid chromatography-tandem mass spectrometry based on enrichment by solid-phase extraction. J. Chromatogr. A.

[35] Wang Z., Broadwater M.H., Ramsdell J.S. (2015). Analysis of diarrhetic shellfish poisoning toxins and pectenotoxin-2 in the bottlenose dolphin (Tursiops truncatus) by liquid chromatography–tandem mass spectrometry. J. Chromatogr. A.

[36] Kruve A., Leito I., Herodes K. (2009). Combating matrix effects in LC/ESI/MS: the extrapolative dilution approach. Anal. Chim. Acta.

[37] Marr J.C., Jackson A.E., Mclachlan J.L. (1992). Occurrence of *Prorocentrum lima*, a DSP toxin-producing species from the Atlantic coast of Canada. J. Appl. Phycol..

[38] Hu T., Marr J., Freitas A.S.W.D., Quilliam M.A., Walter J.A., Wright J.L.C., Pleasance S. (1992). New diol esters isolated from cultures of the dinoflagellates *Prorocentrum lima* and *Prorocentrum concavum*. J. Nat. Prod..

[39] Paz B., Daranas A.H., Cruz P.G., Franco J.M., Pizarro G., Souto M.L., Norte M., Fernández J.J. (2007). Characterisation of okadaic acid related toxins by liquid chromatography coupled with mass spectrometry. Toxicon.

[40] Nagai S., Suzuki T., Nishikawa T. (2011). Differences in the production and excretion kinetics of okadaic acid, dinophysistoxin-1, and pectenotoxin-2 between cultures of *Dinophysis acuminata* and *Dinophysis fortii* isolated from western Japan. J. Phycol..

[41] Ou L.J., Huang X.Y., Huang B.Q., Qi Y.Z., Lu S.H. (2015). Growth and competition for different forms of organic phosphorus by the dinoflagellate *Prorocentrum donghaiense* with the dinoflagellate *Alexandrium catenella* and the diatom *Skeletonema costatum* s.l.. Hydrobiologia.

[42] Witek B., Plinski M. (2000). The first recorded bloom of *Prorocentrum minimum* (Pavillard) Schiller in the coastal zone of the Gulf of Gdańsk. Oceanologia.

[43] Liu R.Y., Liu L., Liang Y.B., Yu J., Xu D.Y., Wei N., Yang L., Guo H. (2016). The distribution, impacts and risks of toxic microalgae and phycotoxins in China. Mar. Environ. Res..

[44] Guillard R.R., Ryther J.H. (1962). Studies of marine planktonic diatoms. I. Cyclotella nana Hustedt and Detonula confervacea (Cleve) gran. Can. J. Microbiol..

