# Supplementary material for: Profiling of Extracellular Toxins Associated with Diarrhetic Shellfish Poison in Prorocentrum lima Culture Medium by High-Performance Liquid Chromatography Coupled with Mass Spectrometry
Source: Toxins (Basel). 2017 Sep 30;9(10):308. doi: 10.3390/toxins9100308 (PMC5666355; doi:10.3390/toxins9100308)
Supplement: Supplementary file 1 [file toxins-09-00308-s001.pdf]

Supplementary Materials: Profiling of extracellular toxins associated with diarrhetic shellfish poison in *Prorocentrum lima* culture medium by high-performance liquid chromatography coupled with mass spectrometry

Lei Pan, Junhui Chen, Huihui Shen, Xiuping He, Guangjiu Li, Xincheng Song, Deshan Zhou and Chengjun Sun

Table S1. Precision of HR-MS instrument.

Toxins	Parameters	1	2	3	4	5	6	Precision RSD (%)
OA	Peak area	34388	34658	36271	35095	35576	33685	2.61
	Retention time (min)	10.142	10.128	10.102	10.097	10.153	10.137	0.22
	Mass error (ppm)	2.11	1.97	2.05	2.12	2.08	2.15	-
DTX1	Peak area	67558	69649	67434	70048	67978	66449	2.03
	Retention time (min)	19.774	19.828	19.683	19.779	19.711	19.838	0.31
	Mass error (ppm)	2.48	2.32	2.44	2.53	2.38	2.52	-

Table S2. Precision of Trap/MS instrument.

Toxins	Parameters	1	2	3	4	5	6	Precision RSD (%)
OA	Peak area	7693095	7507408	7411245	7190027	6798314	7321534	4.20
	Retention time (min)	11.6	11.2	11.4	11.4	11.5	11.4	1.16
DTX1	Peak area	14160877	12957158	13978266	13343835	13316142	13451260	3.33
	Retention time (min)	19.1	19	19.3	19.3	19.4	19.2	0.77

Table S3. Results of linearity and sensitivity for OA and DTX1.

Toxins	Detection ion (*m*/*z*)	Linear range (pg/mL)	Calibration curve	Correlation coefficients (R^2)	LOD (pg/mL)	LOQ (pg/mL)
OA	827.5	0.5625-112.5	$y = 101685x - 164969$	0.9994	0.225	0.5625
DTX1	841.5	0.9375-187.5	$y = 131777x - 108802$	0.9997	0.4725	0.9375

Table S4. Formula and its theoretical precise molecular mass of 93 DSP compounds.

DSP Compounds	Formula	$[M+H]^+$	$[M+NH_4]^+$	$[M+Na]^+$	$[M+K]^+$	$[M-H]^-$	Reference
Norokadanone	$C_{43}H_{66}O_{11}$	759.4678	776.4943	781.4487	797.4237	757.4533	[1],[2]
19-epi-OA	$C_{44}H_{68}O_{13}$	805.4733	822.4998	827.4552	843.4292	803.4587	[1]
OA	$C_{44}H_{68}O_{13}$	805.4733	822.4998	827.4552	843.4292	803.4587	[1],[3],[4]
DTX2	$C_{44}H_{68}O_{13}$	805.4733	822.4998	827.4552	843.4292	803.4587	[1],[4]
DTX2b	$C_{44}H_{68}O_{13}$	805.4733	822.4998	827.4552	843.4292	803.4587	[1]
DTX2c	$C_{44}H_{68}O_{13}$	805.4733	822.4998	827.4552	843.4292	803.4587	[1]
OA methyl ester	$C_{45}H_{70}O_{13}$	819.4889	836.5155	841.4709	857.4448	817.4744	[1],[2]
DTX1	$C_{45}H_{70}O_{13}$	819.4889	836.5155	841.4709	857.4448	817.4744	[1],[3],[4]
35S DTX1	$C_{45}H_{70}O_{13}$	819.4889	836.5155	841.4709	857.4448	817.4744	[1]
Limaol	$C_{47}H_{74}O_{12}$	831.5253	848.5519	853.5072	869.4812	829.5107	[5]
DTX1 methyl ester	$C_{46}H_{72}O_{13}$	833.5046	850.5311	855.4865	871.4605	831.4900	[1]

OA ethyl ester	$C_{46}H_{72}O_{13}$	833.5046	850.5311	855.4865	871.4605	831.4900	[1]
OA glycol	$C_{44}H_{70}O_{15}$	839.4788	856.5053	861.4607	877.4347	837.4642	[1]
27-O-acetyl OA	$C_{46}H_{70}O_{14}$	847.4839	864.5104	869.4658	885.4398	845.4693	[1]
27-O-acetyl DTX1	$C_{47}H_{72}O_{14}$	861.4996	878.5261	883.4815	899.4555	859.4850	[1]
27-O-acetyl OA methyl ester	$C_{47}H_{72}O_{14}$	861.4996	878.5261	883.4815	899.4555	859.4850	[1]
27-O-acetylo-OA methyl ester	$C_{47}H_{73}O_{14}$	863.5146	880.5411	885.4965	901.4705	861.5000	[3]
27-O-acetyl DTX1 methyl ester	$C_{48}H_{74}O_{14}$	875.5151	892.5417	897.4971	913.4710	873.5006	[1]
2-hydroxymethyl-allyl okadaate	$C_{48}H_{74}O_{14}$	875.5151	892.5417	897.4971	913.4710	873.5006	[2]
Tetra-O-methyl OA methyl ester	$C_{49}H_{78}O_{13}$	875.5516	892.5781	897.5335	913.5075	873.5370	[1]
(3E)-5-hydroxy-2-methylene-3-pentenyl OA ester	$C_{50}H_{76}O_{14}$	901.5308	918.5573	923.5127	939.4867	899.5163	[1],[2]
OA C_6-Diol	$C_{50}H_{78}O_{14}$	903.5464	920.5730	925.5284	941.5023	901.5319	[6]
DTX6	$C_{51}H_{76}O_{14}$	913.5308	930.5573	935.5127	951.4867	911.5163	[1],[2]
6-hydroxy-2-methyl-2,4-hexadienyl OA ester	$C_{51}H_{78}O_{14}$	915.5465	932.5730	937.5284	953.5024	913.5319	[1]
OA-D7b	$C_{51}H_{78}O_{14}$	915.5465	932.5730	937.5284	953.5024	913.5319	[1],[7]
5-methylene-6-hydroxy-2-hexen-l-okadaate	$C_{51}H_{78}O_{14}$	915.5465	932.5730	937.5284	953.5024	913.5319	[1],[8]
DTX1 C_6-Diol	$C_{51}H_{80}O_{14}$	917.5621	934.5886	939.5440	955.5180	915.5475	[6]
OA p-nitrophenyl ester	$C_{50}H_{71}NO_{15}$	926.4897	943.5162	948.4716	964.4456	924.4751	[1]
OA-D8a	$C_{52}H_{80}O_{14}$	929.5621	946.5886	951.5440	967.5180	927.5475	[1],[6],[7]
(2E,4E)-7-hydroxy-2-methyl-2,4-heptadienyl OA ester	$C_{52}H_{80}O_{14}$	929.5621	946.5886	951.5440	967.5180	927.5475	[1]
(2E,4E)-7-hydroxy-2-methyl-2,4-heptadien-1-yl OA ester	$C_{52}H_{80}O_{14}$	929.5622	946.5887	951.5441	967.5181	927.5476	[1]
(2E,4Z)-7-hydroxy-2-methyl-2,4-heptadienyl OA ester	$C_{52}H_{80}O_{14}$	929.5622	946.5887	951.5441	967.5181	927.5476	[1]
7-hydroxy-2,4-dimethyl-2,4-heptadienyl OA ester	$C_{53}H_{82}O_{14}$	943.5778	960.6043	965.5597	981.5337	941.5632	[1],[2]
OA-D9b	$C_{53}H_{82}O_{14}$	943.5778	960.6043	965.5597	981.5337	941.5632	[1],[2],[7]
(4Z)-8-hydroxy-2,7-bis(methylene)-4-octenyl OA ester	$C_{54}H_{82}O_{14}$	955.5778	972.6043	977.5597	993.5337	953.5632	[1]
OA-D10a/b	$C_{54}H_{82}O_{14}$	955.5778	972.6043	977.5597	993.5337	953.5632	[1], [9]

5,7-dihydroxy-2,4-bis(methylene)heptyl OA ester	$C_{53}H_{82}O_{15}$	959.5727	976.5992	981.5546	997.5286	957.5581	[1],[2],[3]
DTX1 (R)-phenylglycine methyl ester amide	$C_{54}H_{79}NO_{14}$	966.5574	983.5839	988.5393	1004.5133	964.5428	[1]
DTX1 (S)-phenylglycine methyl ester amide	$C_{54}H_{79}NO_{14}$	966.5574	983.5839	988.5393	1004.5133	964.5428	[1]
OA tetraacetate	$C_{52}H_{76}O_{17}$	973.5156	990.5421	995.4975	1011.4715	971.5010	[1]
5-hydroperoxy-7-hydroxy-2,4-bis(methylene)heptyl OA ester	$C_{53}H_{82}O_{16}$	975.5676	992.5941	997.5495	1013.5234	973.5530	[1],[2]
12:0 OA	$C_{56}H_{90}O_{14}$	987.6404	1004.6669	1009.6223	1025.5963	985.6258	[1]
12:0 DTX2	$C_{56}H_{90}O_{14}$	987.6404	1004.6669	1009.6223	1025.5963	985.6258	[1]
OA 9-anthracenylmethyl ester	$C_{59}H_{78}O_{13}$	995.5516	1012.5781	1017.5335	1033.5075	993.5370	[1]
14:3 OA	$C_{58}H_{88}O_{14}$	1009.6248	1026.6513	1031.6067	1047.5807	1007.6102	[1],[3]
14:3 DTX2	$C_{58}H_{88}O_{14}$	1009.6248	1026.6513	1031.6067	1047.5807	1007.6102	[1]
14:2 OA	$C_{58}H_{90}O_{14}$	1011.6404	1028.6669	1033.6223	1049.5963	1009.6258	[1]
14:2 DTX2	$C_{58}H_{90}O_{14}$	1011.6404	1028.6669	1033.6223	1049.5963	1009.6258	[1]
14:1 OA	$C_{58}H_{92}O_{14}$	1013.6561	1030.6826	1035.6380	1051.6120	1011.6415	[1]
14:1 DTX2	$C_{58}H_{92}O_{14}$	1013.6561	1030.6826	1035.6380	1051.6120	1011.6415	[1]
14:0 OA	$C_{58}H_{94}O_{14}$	1015.6717	1032.6982	1037.6536	1053.6276	1013.6571	[1]
14:0 DTX2	$C_{58}H_{94}O_{14}$	1015.6717	1032.6982	1037.6536	1053.6276	1013.6571	[1]
15:0 OA	$C_{59}H_{96}O_{14}$	1029.6874	1046.7139	1051.6693	1067.6433	1027.6728	[1]
15:0 DTX2	$C_{59}H_{96}O_{14}$	1029.6874	1046.7139	1051.6693	1067.6433	1027.6728	[1]
16:4 OA	$C_{60}H_{90}O_{14}$	1035.6404	1052.6669	1057.6223	1073.5963	1033.6258	[1]
16:4 DTX2	$C_{60}H_{90}O_{14}$	1035.6404	1052.6669	1057.6223	1073.5963	1033.6258	[1]
16:3 OA	$C_{60}H_{92}O_{14}$	1037.6561	1054.6826	1059.6380	1075.6120	1035.6415	[1]
16:3 DTX2	$C_{60}H_{92}O_{14}$	1037.6561	1054.6826	1059.638	1075.612	1035.6415	[1]
16:2 OA	$C_{60}H_{94}O_{14}$	1039.6717	1056.6982	1061.6536	1077.6276	1037.6571	[1]
16:2 DTX2	$C_{60}H_{94}O_{14}$	1039.6717	1056.6982	1061.6536	1077.6276	1037.6571	[1]
16:1 OA	$C_{60}H_{96}O_{14}$	1041.6874	1058.7139	1063.6693	1079.6433	1039.6728	[1]
16:1 DTX2	$C_{60}H_{96}O_{14}$	1041.6874	1058.7139	1063.6693	1079.6433	1039.6728	[1]
16:0 OA	$C_{60}H_{98}O_{14}$	1043.7030	1060.7295	1065.6849	1081.6589	1041.6884	[1]
16:0 DTX2	$C_{60}H_{98}O_{14}$	1043.7030	1060.7295	1065.6849	1081.6589	1041.6884	[1]
17:1 OA	$C_{61}H_{98}O_{14}$	1055.7030	1072.7295	1077.6849	1093.6589	1053.6884	[1]
17:1 DTX2	$C_{61}H_{98}O_{14}$	1055.7030	1072.7295	1077.6849	1093.6589	1053.6884	[1]
16:1 DTX1	$C_{61}H_{98}O_{14}$	1055.7030	1072.7295	1077.6849	1093.6589	1053.6884	[1]
17:0 OA	$C_{61}H_{100}O_{14}$	1057.7187	1074.7452	1079.7006	1095.6746	1055.7041	[1]
17:0 DTX2	$C_{61}H_{100}O_{14}$	1057.7187	1074.7452	1079.7006	1095.6746	1055.7041	[1]
16:0 DTX1	$C_{61}H_{100}O_{14}$	1057.7187	1074.7452	1079.7006	1095.6746	1055.7041	[1]
18:4 OA	$C_{62}H_{94}O_{14}$	1063.6717	1080.6982	1085.6536	1101.6276	1061.6571	[1]
18:4 DTX2	$C_{62}H_{94}O_{14}$	1063.6717	1080.6982	1085.6536	1101.6276	1061.6571	[1]
18:3 OA	$C_{62}H_{96}O_{14}$	1065.6874	1082.7139	1087.6693	1103.6433	1063.6728	[1]
18:3 DTX2	$C_{62}H_{96}O_{14}$	1065.6874	1082.7139	1087.6693	1103.6433	1063.6728	[1]
18:2 OA	$C_{62}H_{98}O_{14}$	1067.7030	1084.7295	1089.6849	1105.6589	1065.6884	[1]
18:2 DTX2	$C_{62}H_{98}O_{14}$	1067.7030	1084.7295	1089.6849	1105.6589	1065.6884	[1]
18:1 OA	$C_{62}H_{100}O_{14}$	1069.7187	1086.7452	1091.7006	1107.6746	1067.7041	[1]

18:1 DTX2	$C_{62}H_{100}O_{14}$	1069.7187	1086.7452	1091.7006	1107.6746	1067.7041	[1]
18:0 OA	$C_{62}H_{102}O_{14}$	1071.7343	1088.7608	1093.7162	1109.6902	1069.7197	[3]
18:0 DTX2	$C_{62}H_{102}O_{14}$	1071.7343	1088.7608	1093.7162	1109.6902	1069.7197	[1]
18:4 DTX1	$C_{63}H_{96}O_{14}$	1077.6874	1094.7139	1099.6693	1115.6433	1075.6728	[1]
18:2 DTX1	$C_{63}H_{100}O_{14}$	1081.7187	1098.7452	1103.7006	1119.6746	1079.7041	[1]
18:0 DTX1	$C_{63}H_{104}O_{14}$	1085.7500	1102.7765	1107.7319	1123.7059	1083.7354	[1]
20:5 OA	$C_{64}H_{96}O_{14}$	1089.6874	1106.7139	1111.6693	1127.6433	1087.6728	[1]
20:5 DTX2	$C_{64}H_{96}O_{14}$	1089.6874	1106.7139	1111.6693	1127.6433	1087.6728	[1]
20:1 OA	$C_{64}H_{104}O_{14}$	1097.7500	1114.7765	1119.7319	1135.7059	1095.7354	[1]
20:1 DTX2	$C_{64}H_{104}O_{14}$	1097.7500	1114.7765	1119.7319	1135.7059	1095.7354	[1]
20:5 DTX1	$C_{65}H_{98}O_{14}$	1103.7030	1120.7295	1125.6849	1141.6589	1101.6884	[1]
22:6 OA	$C_{66}H_{98}O_{14}$	1115.7030	1132.7295	1137.6849	1153.6589	1113.6884	[1]
22:6 DTX2	$C_{66}H_{98}O_{14}$	1115.7030	1132.7295	1137.6849	1153.6589	1113.6884	[1]
22:0 OA	$C_{66}H_{110}O_{14}$	1127.7969	1144.8234	1149.7788	1165.7528	1125.7823	[1]
22:0 DTX2	$C_{66}H_{110}O_{14}$	1127.7969	1144.8234	1149.7788	1165.7528	1125.7823	[1]
22:6 DTX1	$C_{67}H_{100}O_{14}$	1129.7187	1146.7452	1151.7006	1167.6746	1127.7041	[1]

Table S5. Detection of DSP compounds at different culture times of *P. lima*.

Culture time（days）		8	16	22	25
Compound 1	Intracellular	+	+	+	+
	Extracellular	+	+	+	+
Compound 2	Intracellular	+	—	—	+
	Extracellular	+	—	—	+
OA(pg/cell)	Intracellular	+	+	+	+
	Extracellular	+	+	+	+
35S DTX1	Intracellular	+	+	+	+
	Extracellular	+	+	+	+
DTX1(pg/cell)	Intracellular	+	+	+	+
	Extracellular	+	+	+	+
OA-D7b(pg/cell)	Intracellular	+	+	+	+
	Extracellular	+	—	+	—
OA-D9b(pg/cell)	Intracellular	+	+	+	+
	Extracellular	+	—	+	—
OA-D10a/b(pg/cell)	Intracellular	+	+	+	+
	Extracellular	+	—	—	—
5,7-dihydroxy-2,4-dimethylene-heptyl okadaate	Intracellular	+	+	+	—
	Extracellular	+	+	+	+

(+: detected; —：not detected)

1. Evaluation of Matrix Effect

P. minimum (GY-H38) cultured in laboratory also belongs to *Prorocentrum* spp. and we have proved that the algae do not produce DSP toxins, so it was selected as the blank control. The culture medium of blank algae treated by SPE was used to prepared mixed standard solution of OA and DTX1. As a reference, a mixed standard solution of the same concentration level was formulated with methanol. The peak areas of OA and DTX1 were measured by MS/MS analysis. The matrix effect (ME) was calculated according to the following formula: ME(%) = (Ax − As) / As × 100. In the formula, Ax and As is the peak area of the toxin in the mixed standard prepared with the blank algae culture and methanol, respectively. ME below 0% indicated signal suppression, while above 0% revealed signal enhancement.

2. Validation of the Method

2.1. LC-HR-MS

In terms of HRMS identification analysis, parameters including precision, mass deviation and detection limit were evaluated. The mass deviation and precision of the instrument were determined by peak areas, retention times and the measured *m*/*z* value obtained by repeating 6 measurements of mixed standard solution of OA and DTX1. The precision was determined by calculating the relative standard deviation of these measurements. LODs were determined by measurements with successive dilution of matrix-spiked standard solution in blank culture medium until a signal-to-noise (S/N) ratio of 3.

2.2. LC-MS/MS

For the quantitative analysis of multi-stage mass spectrometry, the parameters such as precision, linearity and sensitivity were evaluated. The measurement of mixed standard solution of OA and DTX1 was repeated 6 times to obtain the peak area and retention time data in the EICs, thereby determining the precision of the instrument measurement. The target toxin in the algae culture medium was quantified by the matrix-matched external standard method. The matrix standard curve was plotted with the peak area as the ordinate and the mass concentration as the abscissa. The sensitivity of the method is determined by the LODs and LOQs, which corresponded to the lowest fortification level analyzed from the matrices. The LODs and LOQs were determined by measurements with successive dilution of matrix-spiked standard solution in blank culture medium until a S/N ratios of 3 and 10, respectively.

References

1. Wang, Y.L.; Chen, J.H.; Li, Z.Y.; Wang, S.; Shi, Q.; Cao, W.; Zheng, X.L.; Sun, C.J.; Wang, X.R.; Zheng, L. Determination of typical lipophilic marine toxins in marine sediments from three coastal bays of China using liquid chromatography–tandem mass spectrometry after accelerated solvent extraction. *Mar. Pollut. Bull.* **2015**, *101*, 954−960.
2. Nagai, S.; Suzuki, T.; Nishikawa, T. Differences in the production and excretion kinetics of okadaic acid, dinophysistoxin-1, and pectenotoxin-2 between cultures of *Dinophysis acuminata* and *Dinophysis fortii* isolated from western Japan. *J. Phycol.* **2011**, *47*, 1326−1337.
3. Gerssen, A.; Mulder, P.P.; Boer, J.D. Screening of lipophilic marine toxins in shellfish and algae: Development of a library using liquid chromatography coupled to orbitrap mass spectrometry. *Analyl. Chimica Acta*. **2011**, *685*, 176–185.
4. Hu, T.; Marr, J.; Freitas, A.S.W.D.; Quilliam, M.A.; Walter, J.A.; Wright, J.L.C.; Pleasance, S. New diol esters isolated from cultures of the dinoflagellates *Prorocentrum lima* and *Prorocentrum concavum*. *J. Nat. Prod.* **1992**, *55*, 1631−1637.
5. Yang, A.R.; Lee, S.; Yoo, Y.D.; Kim, H.S.; Jeong, E.J.; Rho, J.R. Limaol: A Polyketide from the Benthic Marine Dinoflagellate *Prorocentrum lima*. *J. Nat. Prod.* **2017**, *80*, 1688−1692.
6. Hu, T.; LeBlanc, P.; Burton, I.W.; Walter, J.A.; McCarron P.; Melanson, J.E.; Strangman, W.K.; Wright, J.L.C. Sulfated diesters of okadaic acid and DTX-1: Self-protective precursors of diarrhetic shellfish poisoning (DSP) toxins. *Harmful Algae* **2017**, *63*, 85–93.
7. Paz, B.; Daranas, A.H.; Cruz, P.G.; Franco, J.M.; Pizarro, G.; Souto, M.L.; Norte, M.; Fernández, J.J. Characterisation of okadaic acid related toxins by liquid chromatography coupled with mass spectrometry. *Toxicon* **2007**, *50*, 225–235.
8. Yasumoto, T.; Seino, N.; Murakami, Y.; Murata, M. Toxins produced by benthic dinoflagellates. *Biol. Bull-US*. **1987**, *172*, 128–131.
9. Orellana, G.; Bussche, J.V.; Meulebroek, L.V.; Vandegehuchte, M.; Janssen, C.; Vanhaecke, L. Validation of a confirmatory method for lipophilic marine toxins in shellfish using UHPLC–HR–Orbitrap MS. *Anal. Bioanal. Chem.* **2014**, *406*, 5303–5312.
